# Biomarkers of Tuberculosis Severity and Treatment Effect: A Directed Screen of 70 Host Markers in a Randomized Clinical Trial

**DOI:** 10.1016/j.ebiom.2017.10.018

**Published:** 2017-10-24

**Authors:** G.B. Sigal, M.R. Segal, A. Mathew, L. Jarlsberg, M. Wang, S. Barbero, N. Small, K. Haynesworth, J.L. Davis, M. Weiner, W.C. Whitworth, J. Jacobs, J. Schorey, D.M. Lewinsohn, P. Nahid

**Affiliations:** a Meso Scale Diagnostics, LLC, Rockville, MD, USA; b University of California, San Francisco, CA, USA; c Yale School of Public Health and Yale School of Medicine, New Haven, CT, USA; d San Antonio VA Medical Center, San Antonio, TX, USA; e Centers for Disease Control and Prevention, Atlanta, GA, USA.; f Pacific Northwest National Laboratory, Richland, WA, USA; g University of Notre Dame, Notre Dame, IN, USA; h Oregon Health and Science University, Portland, OR, USA

**Keywords:** Host immune response, Tuberculosis, Biomarkers, Clinical trials

## Abstract

More efficacious treatment regimens are needed for tuberculosis, however, drug development is impeded by a lack of reliable biomarkers of disease severity and of treatment effect. We conducted a directed screen of host biomarkers in participants enrolled in a tuberculosis clinical trial to address this need. Serum samples from 319 protocol-correct, culture-confirmed pulmonary tuberculosis patients treated under direct observation as part of an international, phase 2 trial were screened for 70 markers of infection, inflammation, and metabolism. Biomarker assays were specifically developed for this study and quantified using a novel, multiplexed electrochemiluminescence assay. We evaluated the association of biomarkers with baseline characteristics, as well as with detailed microbiologic data, using Bonferroni-adjusted, linear regression models. Across numerous analyses, seven proteins, SAA1, PCT, IL-1β, IL-6, CRP, PTX-3 and MMP-8, showed recurring strong associations with markers of baseline disease severity, smear grade and cavitation; were strongly modulated by tuberculosis treatment; and had responses that were greater for patients who culture-converted at 8 weeks. With treatment, all proteins decreased, except for osteocalcin, MCP-1 and MCP-4, which significantly increased. Several previously reported putative tuberculosis-associated biomarkers (HOMX1, neopterin, and cathelicidin) were not significantly associated with treatment response. In conclusion, across a geographically diverse and large population of tuberculosis patients enrolled in a clinical trial, several previously reported putative biomarkers were not significantly associated with treatment response, however, seven proteins had recurring strong associations with baseline radiographic and microbiologic measures of disease severity, as well as with early treatment response, deserving additional study.

## Highlights

• Seven proteins, SAA1, PCT, IL-1β, IL-6, CRP, PTX-3 and MMP-8, show recurring strong associations with markers of baseline tuberculosis disease severity, smear grade and cavitation
• These same proteins were strongly modulated by tuberculosis treatment; and had responses that were greater for patients who culture-converted at 8 weeks.
• Several previously reported putative tuberculosis-associated biomarkers (HOMX1, neopterin, and cathelicidin) were not significantly associated with treatment response in our clinical trial cohort.

The identification of improved biomarkers of tuberculosis treatment effect has been recognized as a key research area by the WHO International Roadmap for Tuberculosis Research because it would accelerate the development of new drugs and regimens, and potentially improve approaches to patient monitoring. Our findings within a rigorously conducted phase 2 clinical trial are that across numerous analyses, seven proteins recur with strong associations with both baseline disease severity and treatment effect. The consistency with which these markers are identified provides impetus for their further study as well as the future pursuit of blood-based treatment monitoring assay development.

## 1 Introduction

The lack of reliable surrogate markers of efficacy has hampered tuberculosis (TB) drug development. The current standard for use as a surrogate endpoint in phase 2 studies remains focused on culture conversion and the most studied is the two month culture status, which has low sensitivity and modest specificity for predicting outcomes after treatment completion.(Horne et al., 2010, Phillips et al., 2016, Phillips et al., 2013, Nahid et al., 2011) Additionally, being dependent on sputum, culture-based monitoring can be challenging to use in extrapulmonary TB, and in patients with paucibacillary disease such as is seen in HIV-coinfected patients and in children.(Wallis et al., 2013, Zumla et al., 2013) Both sputum volume and quality decreases in response to treatment and many patients cannot provide sputum samples for culture after a few weeks of treatment. The development of non-sputum-based biomarkers of treatment response would represent an advance for individual monitoring of TB patients as well as serving as an intermediate marker for use in TB drug development.

As an alternative to sputum-based monitoring, blood-based biomarkers are appealing for several reasons. Blood is relatively easy to collect and, unlike sputum, remains an available source for biomarker measures throughout treatment. Blood-based markers of inflammation and infections are also quantitative, and provide an opportunity to improve predictive power by combining multiple biomarkers into predictive biosignatures. Finally, blood-based markers of treatment response could potentially be translated into point of care assays usable in the field without requiring sophisticated laboratory infrastructure, and biosignatures that at baseline could determine disease severity would also be valuable to clinical trialists and TB care providers for risk stratification purposes, as alternatives to chest radiography, for example. Efforts to identify host biomarkers predictive of treatment outcomes have resulted in the identification of a number of biomarkers that change during TB treatment, albeit most are described in observational cohorts or smaller case-control studies.(Andrade et al., 2013, Azzurri et al., 2005, Coussens et al., 2012, Djoba Siawaya et al., 2009, Djoba Siawaya et al., 2008, Huang et al., 2014, Jayakumar et al., 2015, Lee and Chang, 2003, Mihret et al., 2013, Ostrowski et al., 2006) Such studies acknowledge a variety of limitations including being single center studies, focusing on single markers, using convenience samples, having modest sample sizes, or relying on case-control designs.

In the work described herein, serum collected in a standardized manner in a rigorously conducted clinical trial sponsored by the CDC-funded TB Trials Consortium, was used to evaluate the efficacy and safety of a rifapentine-based regimen for drug-susceptible TB (Dorman et al., 2012) Study participants received treatment under direct observation, thereby enhancing and carefully measuring adherence; an important benchmark when assessing biomarkers of treatment effect. We measured the concentration, and change in concentration, of 70 potential biomarkers associated with inflammatory, antimicrobial, T-cell and acute phase responses to bacterial infections, and with tissue remodeling at infection sites. These biomarkers were selected because they have been published as indicators of TB disease and the clinical trial samples provided an opportunity to reassess these associations across diverse international sites (Andrade et al., 2013, Azzurri et al., 2005, Coussens et al., 2012, Djoba Siawaya et al., 2009, Djoba Siawaya et al., 2008, Huang et al., 2014, Jayakumar et al., 2015, Lee and Chang, 2003). Access to clinical trial-quality data allowed us to account for changes in biomarker levels across disease phenotypes, regimens, and geographic regions, in order to identify biomarkers associated with treatment response.

## 2 Methods

### 2.1 Study Design and Population

The parent study was a CDC-sponsored clinical trial, Tuberculosis Trials Consortium (TBTC) Study 29 (ClinicalTrials.gov Identifier NCT00694629). This was a randomized phase 2 trial, comparing the antimicrobial activity and safety of a standard daily regimen containing rifampin, to that of the experimental regimen with daily rifapentine (10 mg/kg), both given with isoniazid, pyrazinamide and ethambutol to adults with smear-positive, culture-confirmed pulmonary TB. All TB treatment was administered 5 days/week and directly observed. All participants underwent HIV testing. Information regarding the design, conduct, and results of TBTC Study 29 has been published (Dorman et al., 2012). Out of a total of 531 participants in the parent study, 389 consecutively enrolled protocol-correct participants (rather than the modified intention-to-treat population, since adherence may not be optimal in this population) were included in this biomarker study. Of 389 participants, 319 had paired baseline and week 8 serum samples available for biomarker testing. The parent trial excluded patients if they had received > 5 days of TB treatment in the preceding 6 months, however, treatment of < 5 days was permissible, and was noted in 60% of study participants; the median number of days of treatment prior to enrollment was 2 days, (IQR of 0 to 4 days). Detailed clinical, radiographic and microbiologic data including sputum culture status at 8 weeks and 12 weeks (determined on both liquid and solid media) were collected in a standardized manner as part of the parent clinical trial and used in biomarker analyses. Written informed consent was obtained from all study participants for collection of serum for TB-related research. In addition, the institutional review board at University of California, San Francisco (UCSF) approved this ancillary study to assess putative biomarkers of treatment response (approval #12-10360).

### 2.2 Specimen Collection

Blood was collected at enrollment (baseline), and after 8 weeks of combination drug therapy, using Becton Dickinson Serum Separator Tubes (BD Vacutainer® SST™ Tube, BD Diagnostics, Franklin Lakes, NJ, USA). BD Vacutainer® SST™ Tubes were centrifuged within 2 h of collection and processed according to manufacturer recommendations. Collection, processing and storage of sera was conducted according to a standardized manual of operating procedures that has been confirmed to provide quality samples free of processing errors (Nahid et al., 2014). Serum was aliquoted on site, frozen at − 70 °C, and batch-shipped on dry ice.

### 2.3 Multiplexed Immunoassays

A total of 70 biomarkers were measured in 14 multiplexed assay panels using a sandwich immunoassay format (proteins) or a competitive immunoassay format (neopterin), using electrochemiluminescence (ECL) detection (Debad et al., 2004). The ECL assays employed consumables and instrumentation from Meso Scale Diagnostics, LLC (MSD). The assay components for each panel included a MSD MULTI-ARRAY® 96-well plate having an array of capture antibodies in each well, a set of labeled detection antibodies for each analyte in the panel (labeled with the MSD SULFO-TAG™ ECL label), a combined calibration standard containing a mixture of the target analytes, an assay diluent and a detection antibody diluent. For the neopterin competitive assay, a labeled neopterin analog was used in the place of the labeled detection antibody. In total, 14 biomarker panel assays were tested, six MSD commercial kits and eight custom assay panels that were newly developed for this study (see Supplemental materials on Assay Methods).

### 2.4 Clinical Sample Tests

MSD received 500 μL of each sample at their core facility (Gaithersburg, MD) where assays were conducted with investigators and technicians blinded to participant data. Each sample was tested in duplicate with each of the 70 assays. Concentrations were reported as the average value of the duplicate measurements; values below the LOD were assigned a concentration equal to the LOD. CVs were determined for the biomarker levels measured in the control samples run on each assay plate; the median control CV (and IQR) across the different assays was 10% (9%–13%).

### 2.5 Statistical Methods

Statistical analysis was conducted using the R statistical programming language (version 3.2.3). Analysis of biomarkers used log_10_-transformed concentrations. The Student's *t*-test was used for comparing means across groups. Linear regression (using the “lm” function in R) was used to determine the association of biomarker concentrations with clinical variables and to adjust for potential covariates. The two-sided t-statistic was used to determine the significance of regression coefficients with a threshold of *p* < 0.05 taken as statistically significant. To account for multiple testing, we performed a Bonferroni correction for the number of tests applied in each analysis.

Linear regression was used to identify associations of log_10_-transformed baseline biomarker levels with baseline clinical indicators of disease severity at baseline: smear grade (grade = 1 vs. grade ≥ 2), chest radiograph status (no cavities vs cavities; cavities ≤ 4 cm vs cavities > 4 cm; and extent of lung involvement < 50% vs lung involvement > 50%) and MGIT time-to-detection (≤ 5 days vs > 5 days). Two linear models were employed: (i) an unadjusted model and (ii) a model that adjusted for potential demographic and clinical covariates (gender, age, body mass index, HIV status, study location (African vs. non-African) and study arm).

For each biomarker, the treatment effect for a given participant was calculated as C_8_/C_0;_ the ratio of the biomarker levels at week 8 (C_8_; concentration after treatment) relative to week 0 (C_0_; concentration at baseline). Linear regression was used to examine the significance of the association of the log_10_-transformed treatment effect with culture conversion. Three models were used: (i) an unadjusted model; (ii) a model that adjusted for demographic covariates, and (iii) an adjusted model that included indicators of baseline disease severity (as described above).

We also investigated whether biomarker combinations were predictive of week-8 culture status. The primary methodology employed was L1 penalized (Lasso) logistic regression (R package glmnet), with random forests also being deployed (R package random ForestSRC). (Hastie et al., 2009).

## 3 Results

The demographic and clinical characteristics of the 319 protocol-correct trial participants included in biomarker analyses are shown in Table 1. Three patients (1%) remained sputum culture positive at 16 weeks and were adjudicated to have failed treatment.

**Table 1:** Demographic and clinical characteristics of the study population.

	No. patients	Number with stable sputum conversion on solid and liquid media		
	(% of total)	(% of category)		
Characteristic		At 8 weeks	At 12 weeks	At 16 weeks
All subjects	319 (100%)	209 (66%)	264 (83%)	316 (99.1%)
Gender				
Female	107 (34%)	86 (80%)	93 (87%)	105 (98.1%)
Male	212 (66%)	123 (58%)	171 (81%)	211 (99.5%)
Age				
0–20	17 (5%)	13 (76%)	16 (94%)	17 (100.0%)
21–40	203 (64%)	128 (63%)	169 (83%)	203 (100.0%)
41–60	80 (25%)	54 (68%)	63 (79%)	78 (97.5%)
61	19 (6%)	14 (74%)	16 (84%)	18 (94.7%)
Body mass index				
BMI ≤ 18.5	97 (30%)	56 (58%)	80 (82%)	95 (97.9%)
BMI > 18.5	222 (70%)	153 (69%)	184 (83%)	221 (99.5%)
Location				
N. America	109 (34%)	85 (78%)	99 (91%)	107 (98.2%)
Spain	24 (8%)	17 (71%)	19 (79%)	24 (100.0%)
S. Africa	64 (20%)	44 (69%)	54 (84%)	64 (100.0%)
Uganda	122 (38%)	63 (52%)	92 (75%)	121 (99.2%)
HIV status				
Negative	284 (89%)	189 (67%)	235 (83%)	282 (99.3%)
Positive	35 (11%)	20 (57%)	29 (83%)	34 (97.1%)
Baseline smear				
Low	114 (36%)	91 (80%)	102 (89%)	114 (100.0%)
High	205 (64%)	118 (58%)	162 (79%)	202 (98.5%)
Baseline chest x-Ray				
No cavity	115 (36%)	84 (73%)	98 (85%)	115 (100.0%)
Cavity ≤ 4 cm	80 (25%)	54 (68%)	70 (88%)	79 (98.8%)
Cavity > 4 cm	124 (39%)	71 (57%)	96 (77%)	122 (98.4%)
Baseline MGIT960				
TTD < = 5 days	82 (26%)	45 (55%)	64 (78%)	79 (96.3%)
TTD > 5 days	237 (74%)	164 (69%)	200 (84%)	237 (100.0%)
Study arm				
Rifampin	151 (47%)	93 (62%)	124 (82%)	151 (100.0%)
Rifapentine	168 (53%)	116 (69%)	140 (83%)	165 (98.2%)

MGIT960 TTD refers to the time-to-detection for baseline liquid mycobacterium growth indicator tube system (MGIT) 960 cultures. Sputum conversion indicates negative sputum culture on both solid and liquid media at the specified time point.

Using multiplexed ECL assays, we quantified the serum levels of 69 proteins and one metabolic marker. For a majority of these biomarkers (62 of 70), the level was greater than twice the limit of detection for at least 75% of the samples (Supplemental Table 1).

A small subset (8 of 70) provided levels above this threshold for < 25% of the samples. We excluded these assays – GM-CSF, IL-1α, IL-2, IL-4, IL-12p70, IL-13, IFN-2α and IFNβ – from further analysis.

### 3.1 Baseline Biomarkers Associated With Baseline Clinical Characteristics

**Fig. 1:**
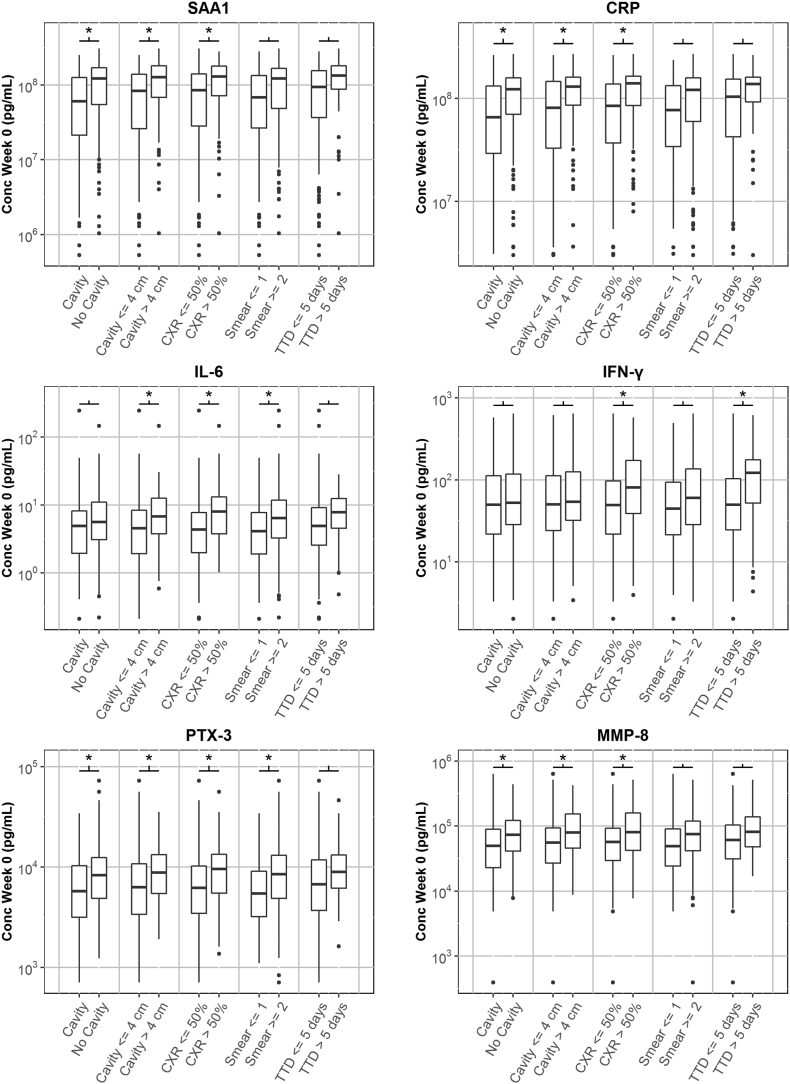
Box and whisker plots showing the baseline levels of six biomarkers as a function of five indicators of baseline disease state: presence of cavities in chest x-ray, presence of cavities > 4 cm, lung involvement > 50%, smear grade (≤ 1 vs ≥ 2) and time-to-detection (≤ 5 days vs > 5 days) for MGIT cultures. The asterisks indicate that the difference between two groups is significant (*p* < 0.05/62 assays = 0.0008).

Linear models were used to measure the association of biomarker levels with disease severity at baseline, with and without correction for demographic covariates. Table 2, Table 3 show the biomarkers that had a coefficient significantly different than 0 in at least one of the models. For the unadjusted model, we found biomarkers with significant associations with each of the indicators. Adjustment for demographic covariates tended to decrease the significance of the associations. Even after adjustment, however, some biomarkers retained significant associations for all indicators except MGIT time-to-detection. Fig. 1 shows the baseline biomarker levels against disease classification group, for six biomarkers (SAA1, CRP, IL-1β, IL-6, PTX-3 and MMP-8) that tended to have the most significant increases in baseline levels with increases in disease severity indicators.

**Table 2:** Association of baseline biomarker levels with baseline chest radiograph characteristics.

[A]						
Biomarkers associated with presence of cavities at baseline						
	Unadjusted model			Adjusted (demographics)		
Assay	Est (95%CI)	P value	AUC	Est (95%CI)	P value	AUC
SAA1	1.97 (1.71, 2.28)	0.0000	0.65	1.81 (1.58, 2.08)	0.0000	0.65
CRP	1.74 (1.55, 1.94)	0.0000	0.65	1.60 (1.44, 1.78)	0.0000	0.64
IL-1β	1.56 (1.37, 1.77)	0.0005	0.62	1.42 (1.26, 1.58)	0.0023	0.61
MMP-8	1.54 (1.38, 1.71)	0.0001	0.63	1.42 (1.28, 1.57)	0.0006	0.61
PTX-3	1.39 (1.27, 1.53)	0.0004	0.61	1.27 (1.17, 1.39)	0.0040	0.61
MMP-9	1.37 (1.27, 1.48)	0.0001	0.63	1.31 (1.22, 1.42)	0.0004	0.61
sICAM-1	1.17 (1.12, 1.22)	0.0004	0.61	1.12 (1.08, 1.16)	0.0023	0.60

[B]						
Biomarkers associated with cavities > 4 cm at baseline						
	Unadjusted model			Adjusted (demographics)		
Assay	Est (95%CI)	P value	AUC	Est (95%CI)	P value	AUC
SAA1	1.90 (1.65, 2.19)	0.0000	0.65	1.62 (1.41, 1.86)	0.0005	0.62
IL-1β	1.77 (1.57, 2.00)	0.0000	0.65	1.48 (1.32, 1.65)	0.0006	0.62
CRP	1.67 (1.50, 1.87)	0.0000	0.64	1.45 (1.31, 1.62)	0.0004	0.61
IL-6	1.59 (1.41, 1.79)	0.0001	0.62	1.35 (1.20, 1.52)	0.0093	0.60
MMP-8	1.55 (1.39, 1.73)	0.0000	0.64	1.35 (1.22, 1.49)	0.0036	0.60
PTX-3	1.39 (1.27, 1.52)	0.0003	0.62	1.20 (1.10, 1.31)	0.0306	0.58
sICAM-1	1.30 (1.24, 1.35)	0.0000	0.70	1.21 (1.17, 1.25)	0.0000	0.68
E-Selectin	1.29 (1.21, 1.37)	0.0001	0.63	1.22 (1.15, 1.30)	0.0017	0.61
IL-2R	1.27 (1.20, 1.35)	0.0001	0.64	1.18 (1.12, 1.25)	0.0022	0.61
LBP	1.26 (1.18, 1.34)	0.0002	0.63	1.18 (1.11, 1.25)	0.0065	0.61
TNF-RI	1.17 (1.12, 1.22)	0.0003	0.62	1.13 (1.08, 1.17)	0.0045	0.61

[C]						
Biomarkers associated with TB extent of disease on baseline chest radiograph (> 50% lung involvement)						
	Unadjusted model			Adjusted (demographics)		
Assay	Est (95%CI)	P value	AUC	Est (95%CI)	P value	AUC
IL-1β	2.42 (2.15, 2.72)	0.0000	0.73	2.00 (1.79, 2.23)	0.0000	0.70
IL-6	1.89 (1.67, 2.13)	0.0000	0.67	1.56 (1.38, 1.76)	0.0002	0.64
SAA1	1.87 (1.62, 2.16)	0.0000	0.65	1.55 (1.34, 1.79)	0.0025	0.62
PCT	1.78 (1.57, 2.02)	0.0000	0.65	1.46 (1.28, 1.66)	0.0039	0.61
IP-10	1.67 (1.50, 1.87)	0.0000	0.66	1.49 (1.34, 1.67)	0.0003	0.64
VEGF	1.66 (1.52, 1.82)	0.0000	0.68	1.49 (1.37, 1.63)	0.0000	0.65
IFN-γ	1.63 (1.44, 1.85)	0.0001	0.63	1.42 (1.26, 1.60)	0.0031	0.60
CRP	1.62 (1.45, 1.81)	0.0000	0.65	1.33 (1.19, 1.49)	0.0097	0.59
I-TAC	1.59 (1.40, 1.80)	0.0002	0.62	1.28 (1.15, 1.42)	0.0245	0.59
CXCL9	1.57 (1.44, 1.72)	0.0000	0.66	1.43 (1.31, 1.56)	0.0000	0.64
MIP-3α	1.55 (1.38, 1.73)	0.0001	0.63	1.40 (1.26, 1.55)	0.0016	0.62
IL-2R	1.52 (1.44, 1.61)	0.0000	0.73	1.36 (1.29, 1.44)	0.0000	0.70
MMP-8	1.48 (1.33, 1.65)	0.0003	0.61	1.24 (1.12, 1.38)	0.0421	0.56
E-Selectin	1.48 (1.39, 1.57)	0.0000	0.70	1.38 (1.29, 1.47)	0.0000	0.68
PTX-3	1.46 (1.33, 1.59)	0.0000	0.64	1.20 (1.10, 1.31)	0.0357	0.58
Osteopontin	1.41 (1.33, 1.48)	0.0000	0.71	1.26 (1.20, 1.33)	0.0000	0.67
MMP-1	1.36 (1.25, 1.49)	0.0005	0.62	1.19 (1.09, 1.30)	0.0457	0.57
uPAR	1.34 (1.28, 1.41)	0.0000	0.69	1.28 (1.22, 1.34)	0.0000	0.67
sICAM-1	1.34 (1.29, 1.40)	0.0000	0.73	1.24 (1.20, 1.29)	0.0000	0.71
MMP-9	1.32 (1.22, 1.42)	0.0005	0.62	1.18 (1.09, 1.28)	0.0364	0.57
Granzyme-B	1.28 (1.20, 1.37)	0.0003	0.63	1.17 (1.10, 1.25)	0.0148	0.60
LBP	1.27 (1.19, 1.35)	0.0002	0.63	1.17 (1.10, 1.25)	0.0114	0.60
TNF-RII	1.21 (1.15, 1.28)	0.0005	0.62	1.12 (1.06, 1.19)	0.0387	0.58
Neopterin	1.20 (1.15, 1.26)	0.0000	0.63	1.20 (1.15, 1.25)	0.0001	0.63
TNF-RI	1.19 (1.14, 1.25)	0.0000	0.64	1.12 (1.08, 1.17)	0.0077	0.60
LBP	1.27 (1.19, 1.35)	0.0002	0.63	1.17 (1.10, 1.25)	0.0114	0.60

The estimated association (Est) is 10^coeff^, where coeff is the coefficient of a linear model for the effect of the indicated baseline radiographic characteristics on log_10_ transformed baseline biomarker levels. The Est value can be interpreted as the expected factor change in baseline biomarker level for patients with [A] cavities relative to patients with no cavities; [B] cavities > 4 cm relative to patients with cavities < 4 cm; [C] lung involvement > 50% relative to patients with lung involvement < 50%. The tables show values for an unadjusted model as well as a model that is adjusted for demographic covariates (gender, age, BMI, HIV status, region (Africa vs. Not-Africa) and study arm).The AUC value for ROC curves is a non-parametric indicator of effect size, comparing the distributions of biomarker ratios for the two outcome classes (either unadjusted or adjusted for the covariates). Only biomarkers with statistically significant associations (*p* < 0.05/62 assays = 0.0008) in at least one of the models are shown.

**Table 3:** Association of baseline biomarker levels with baseline microbiological characteristics.

[A]						
Smear grade > 1 at baseline						
	Unadjusted model			Adjusted (demographics)		
Assay	Est (95%CI)	P value	AUC	Est (95%CI)	P value	AUC
SAA1	1.68 (1.45, 1.95)	0.0004	0.62	1.52 (1.32, 1.74)	0.0027	0.60
IL-1β	1.57 (1.38, 1.78)	0.0004	0.62	1.39 (1.24, 1.56)	0.0033	0.60
IL-6	1.52 (1.34, 1.72)	0.0008	0.62	1.37 (1.22, 1.54)	0.0060	0.59
PTX-3	1.49 (1.36, 1.63)	0.0000	0.64	1.37 (1.26, 1.48)	0.0002	0.63
TNF-RI	1.19 (1.14, 1.24)	0.0001	0.62	1.15 (1.11, 1.20)	0.0006	0.61

[B]						
MGIT time-to-detection						
	Unadjusted model			Adjusted (demographics)		
Assay	Est (95%CI)	P value	AUC	Est (95%CI)	P value	AUC
IFN-γ	1.82 (1.56, 2.12)	0.0001	0.67	1.48 (1.28, 1.70)	0.0062	0.63
IL-15	1.27 (1.19, 1.34)	0.0001	0.67	1.19 (1.12, 1.27)	0.0038	0.63

The estimated association (Est) is 10^coeff^, where coeff is the coefficient of a linear model for the effect of the indicated baseline microbiological characteristics on log_10_ transformed baseline biomarker levels. The Est value can be interpreted as the expected factor change in baseline biomarker level for patients with [A] baseline smear grade > 1 relative to patients with smear grade < 1; [B] baseline MGIT time-to-detection ≤ 5 days relative to patients with MGIT time-to-detection > 5 days. The tables show values for an unadjusted model as well as a model that is adjusted for demographic covariates (gender, age, BMI, HIV status, region (Africa vs. Not-Africa) and study arm). The AUC value for ROC curves is a non-parametric indicator of effect size, comparing the distributions of biomarker ratios for the two outcome classes (either unadjusted or adjusted for the covariates). Only biomarkers with statistically significant associations (*p* < 0.05/62 assays = 0.0008) in at least one of the models are shown.

The analysis of baseline biomarker levels was repeated after stratification for HIV status. Limiting analysis to only HIV negative patients did not significantly change the identified biomarkers or the strengths of the measured associations with baseline clinical characteristics (data not shown). The study was not sufficiently powered for identifying biomarkers associated with baseline clinical characteristics for the HIV positive subjects, however, qualitatively the association of the two acute phase proteins SAA1 and CRP with baseline clinical characteristics appeared to be much weaker in the HIV positive subjects relative to the HIV negative subjects. For example, for HIV negative subjects, SAA1 was 2.09-fold higher (CI: 1.79 to 2.43) in subjects with cavities at baseline and 1.77-fold higher (CI: 1.51 to 2.07) in subjects with sputum smear grades of 2 or higher, while for HIV positive subjects the measured increases in SAA1 associated with these two indicators were only 1.44-fold (CI: 0.99 to 2.11) and 1.16-fold (0.78 to 1.71), respectively.

### 3.2 Effect of Treatment on Biomarker Levels

**Fig. 2:**
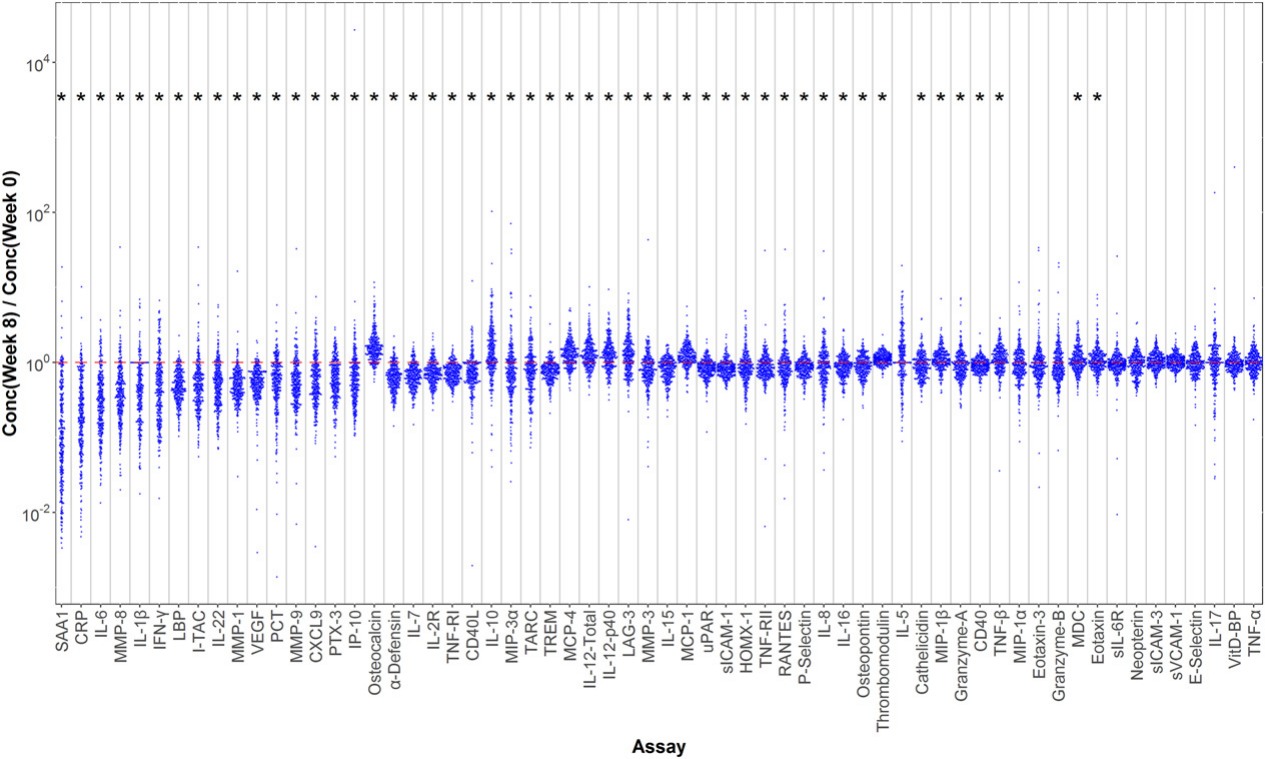
Effect of TB treatment on biomarker levels. The plot shows the ratio of post-treatment (week 8) to baseline (week 0) biomarker levels for each biomarker in each study subject. The assays are ordered based on the magnitude of the treatment effect. Asterisks indicate the effect is statistically significant (p < 0.05/62 assays = 0.0008).

Fig. 2 shows the treatment effect for all biomarkers across all patients. Qualitatively, a large number of biomarkers appear to be influenced by treatment and have a preponderance of points with ratios above or below one. The spread in the effect for different subjects, however, is large and for some assays covers more than a factor of ten. Biomarkers with significant treatment effects were selected based on two criteria (Supplemental Table 2). First, the geometric average treatment effect was significantly different from one. Second, the inter-quartile range (IQR) did not include the null result (i.e., C_8_/C_0_ = 1). Most of the selected biomarkers (23 of 26) exhibited, on average, decreases in levels with treatment; only three biomarkers increased with treatment. Three of the largest average decreases with treatment were observed for the acute phase proteins SAA1, CRP and LBP, with the largest average decrease (~ ten-fold) exhibited by SAA1. Large decreases were also observed for cytokines associated with host inflammatory response (IL-6, IL-1β and IL-22), and biomarkers of tissue reorganization and repair (MMP-1, MMP-8 and VEGF). The largest increase in biomarker levels with treatment was observed for osteocalcin (~ 1.6-fold), a bone-derived biomarker with a role in bone synthesis and glucose metabolism.

Stratification by HIV status indicated that the markers showing changes with treatment and the sizes of the measured treatment effects were not significantly different for the HIV positive and negative subgroups (data not shown). One exception was LAG-3. For the HIV negative subjects, LAG-3 demonstrated a relatively modest 1.18-fold increase in average biomarker level with treatment (CI: 1.10 to 1.27). However, in the HIV positive group, a significantly larger 1.90-fold increase was observed (CI: 1.64 to 2.39).

### 3.3 Biomarkers Associated With Microbiologic Treatment Response

**Fig. 3:**
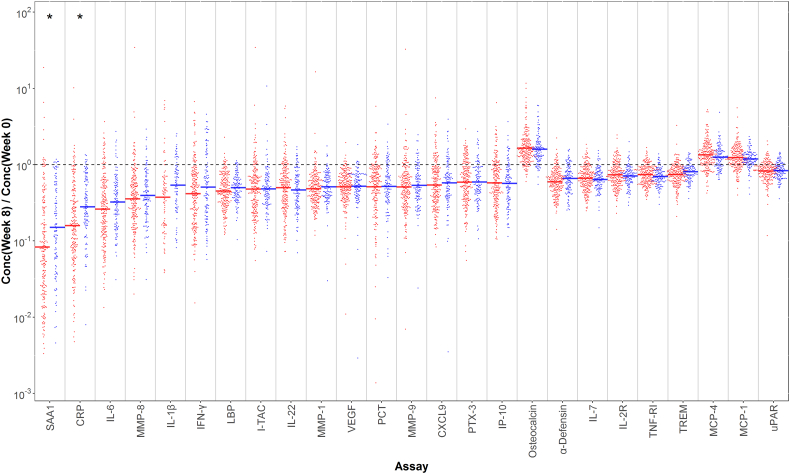
Association of biomarker treatment effect with sputum culture conversion status at week 8. The plot shows the ratio of post-treatment (week 8) to baseline (week 0) biomarker levels for the 25 biomarkers with the strongest treatment effects (the top 25 assays in Supplemental Table 2). Points are separated and colored based on sputum culture conversion status at week 8 (blue = converted, red = non-converted). For each biomarker and group, a horizontal line segment indicates the median biomarker value. The biomarkers with a significant association with culture status (p < 0.05/62 biomarkers = 0.0008) are marked with an asterisk.

Fig. 3 shows the ratio of week-8 and week-0 biomarker levels for the top 25 biomarkers listed in Supplemental Table 2 across all patients, coloring the points based on culture conversion status at week 8. Qualitatively, the change in biomarker levels with treatment tends to be greater for the patients that achieve culture conversion, especially for the biomarkers that are most affected by treatment. However, there is considerable overlap in the distributions.

The associations of biomarker treatment response with week-8 culture status for the six biomarkers with the strongest changes after treatment (the top six biomarkers in Supplemental material Table 2) are shown in Supplemental Table 3. Three biomarkers with strong responses to treatment (SAA1, CRP and IL-1β) were the only biomarkers that also showed an association with week-8 culture status in at least one of the models that met our criteria for significance. These biomarkers also showed the expected directionality for the effect; the mean ratio of week-8 to baseline biomarker levels for SAA and CRP were almost two-fold higher for converted patients, relative to non-converted patients. The three additional markers listed in Supplemental Table 3 (IL-6, MMP-8 and IFN-γ) also showed an apparent association with week-8 culture status in the expected direction, but did not achieve our criteria for significance. For four of the six biomarkers (SAA1, IL-1β, IL-6 and MMP-8), the strength of the association increased and the *p* value decreased with the addition of covariates to the model. Analyzing the HIV positive and negative subjects separately did not change the identity of the biomarkers with the strongest associations with week-8 culture status.

Whereas week-8 culture conversion was associated with ~ two-fold increase in the effect of treatment on levels of SAA1 and CRP, the biomarkers with the largest effect sizes, the size of this effect was still small relative to the overall variation of the treatment effect within each group. For this reason, the predictive ability of any individual assay for week-8 culture status is likely to be relatively weak. The area under curve (AUC) values for receiver operating characteristic (ROC) analysis can be used as a non-parametric indicator of effect size. The AUC values for SAA1 and CRP were relatively low, ranging from 0.62 to 0.65 depending on the model used.

**Fig. 4:**
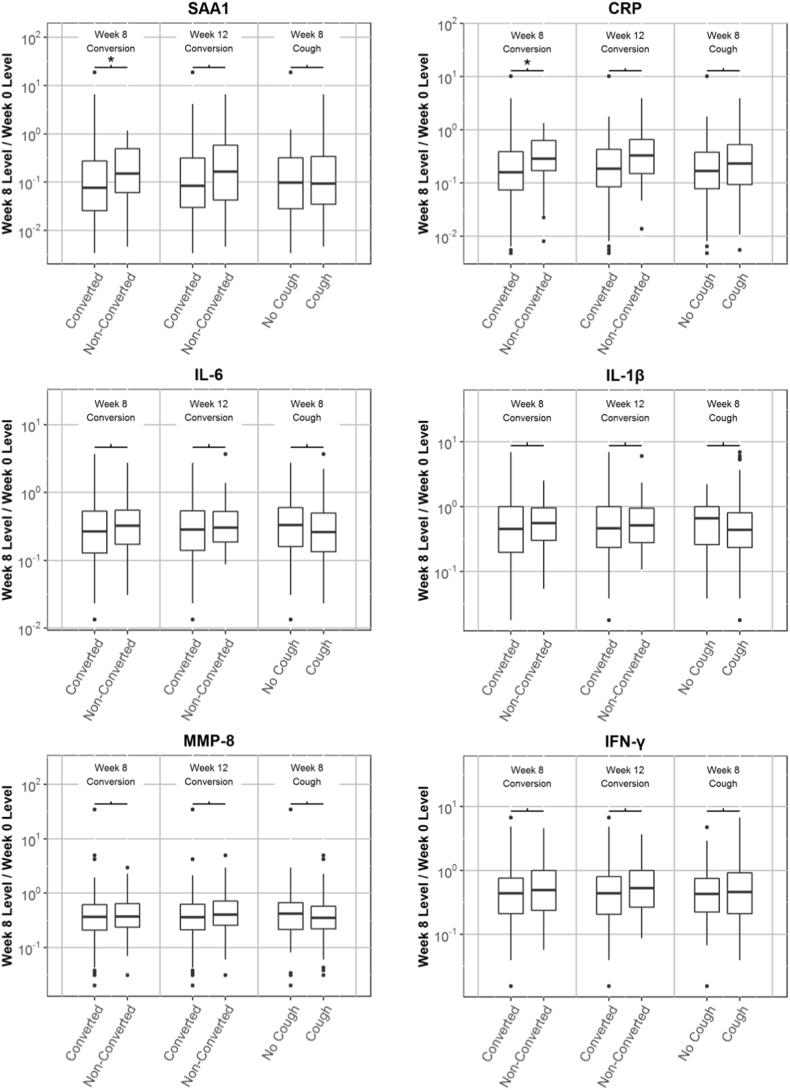
Box and whisker plots showing the magnitude of the treatment effect for six biomarkers (the ratio of week 8 and week 0 biomarker levels) after grouping subjects according to three outcome variables: week 8 culture conversion status, week 12 culture conversion status, and presence/absence of cough at 8 weeks. The asterisks indicate that the difference between two groups is significant (p < 0.05/62 assays = 0.0008).

For the six biomarkers with the strongest response to treatment, we compared the strength of the association of biomarker response with week-8 culture status, week-12 culture status and week-8 cough status (Fig. 4). Analysis of the association of the biomarkers with week-12 culture status showed trends that were similar to those described above for week-8 culture status, although the strength of the associations was weaker and the *p* values did not meet our criteria for significance. For example, the increase in treatment response (unadjusted for covariates) with week-8 culture conversion was 1.7-fold (*p* = 0.0001) for CRP and 1.8-fold (*p* = 0.0009) for SAA1, but the association with week-12 culture conversion was 1.6-fold (*p* = 0.040) for CRP and 1.6-fold (*p* = 0.010) for SAA1.

Association of the biomarker response to treatment with week-8 cough status was weak. As with culture status, the largest increases in the biomarker response to treatment with absence of cough at 8 weeks (unadjusted for covariates) were observed for SAA1 (1.2-fold, *p* = 0.37) and CRP (1.3-fold, *p* = 0.048).

Use of biomarker combinations as predictors of week-8 culture status did not reveal any compelling signatures. The modest AUC values reported above for individual biomarkers were only incrementally improved when signatures were derived using L1 penalized logistic regression. For example, using cross-validated AUC as a predictive model performance metric and invoking the “1 SE rule” for determining model size and restricting analysis to HIV negative subjects yields a set of four markers (CRP, IL_10, MMP_3 and IFN_g). However, the attendant cross-validated AUC is only 0.63. While a 13 marker model was optimal, achieving a value of 0.66, the variability associated with this estimate make the stability of the corresponding signature questionable. Similarly, random forest-based classification did not produce improved predictive performance. Analysis of biomarker associations with treatment failure was not feasible as only three trial participants (1%) failed treatment.

## 4 Discussion

In this study, we identified biomarkers associated with radiographic and microbiologic measures of severity, as well as biomarkers associated with treatment response. Seven baseline biomarkers were significantly associated with the presence or absence of cavities at enrollment. The strongest effect was observed for the acute phase protein SAA1 (serum amyloid protein A), which showed a two-fold increase at baseline in patients with cavities, as compared to patients without cavities. The other proteins associated with cavitation included the inflammatory cytokine IL-1β, the tissue-remodeling factor MMP-8 (matrix metalloproteinase 8) and the cell migration factor sICAM-1 (soluble intercellular cell adhesion molecule 1). Interestingly, the number of biomarkers associated with baseline TB disease increased with increasing disease severity: seven biomarkers were associated with the presence of any cavities, 11 biomarkers associated with the presence of cavities larger than 4 cm, and 26 biomarkers associated with ≥ 50% of lung involvement. The associations between serum biomarkers and severity of lung injury is plausible given production of these markers in the lung has also been reported. For instance, SAA1 is expressed in the lung, and has been associated with lung injury as well as lung cancer (Lopez-Campos et al., 2013, Baba et al., 1992). Similarly, the collectin Pentraxin 3 can both be produced in lung endothelial cells in response to IL-1 (Pauwels et al., 2010) or in lung fibroblasts in response to TNF-α (Mantovani et al., 2013) and is associated with pulmonary fibrosis (Pilling et al., 2015). VEGF is expressed by epithelial cells as well as by *M. tuberculosis*-infected macrophages, and has been implicated in granuloma-associated angiogenesis (Polena et al., 2016). Finally, elevated levels of alpha-defensin are associated with pulmonary fibrosis (Mukae et al., 2002, Sakamoto et al., 2015a, Sakamoto et al., 2015b). Taken together, these data suggest that selected markers measured in serum reflect both the burden of microbial infection and the degree of lung injury.

Biomarker associations with baseline measures of microbiologic burden, namely smear grade and MGIT time-to-detection, were also evaluated. We identified five biomarkers, SAA1, IL-1β, IL-6, PTX-3 and TNF-RI, that had significant associations with smear grade. These biomarkers were also among those that had the strongest associations with more extensive disease on chest radiograph (described above). Two additional biomarkers, IFN-γ and IL-15, were associated with MGIT time-to-detection, and were distinct from the five markers associated with smear grade. The identification of serum markers with strong associations with baseline bacillary burden raises the possibility for rapid point-of-care tests that could inform decisions at the time of treatment initiation in terms of regimen composition and duration, allowing for potential individualization of care.

On treatment, the majority of biomarkers declined significantly, suggesting aberrant levels at baseline that return to normal as the disease is treated, bacterial burden declines and lung injury resolves. Importantly, we also found that several previously reported putative TB-associated biomarkers (HOMX1, neopterin, and cathelicidin) were not significantly associated with treatment response. The biomarkers with the strongest treatment responses were SAA1, CRP, IL-6, MMP-8, and IL-1β, all of which were also linked to disease severity and measures of baseline bacillary burden, providing additional evidence that these markers are indicators of disease state at time of diagnosis and during the course of treatment. Treatment also decreased alpha-defensin levels, not previously reported in TB, although predicted by gene expression studies (Maertzdorf et al., 2012). Plasma heme oxygenase-1 (HOMX1) has previously been reported to have a strong response to TB treatment, (Andrade et al., 2013) but we found little change in HOMX1 levels with treatment. Neopterin has also been reported to decrease significantly with treatment; (Turgut et al., 2006) in our study we did not see a large change in neopterin, but this result may be related to differences in timing of sample collection, as prior reports found that neopterin levels return to baseline levels only after six months of treatment. Whereas plasma protein 10 (IP-10)/CXCL10 has previously been reported to be useful in monitoring patients in smaller studies, (Azzurri et al., 2005, Wergeland et al., 2015, Kabeer et al., 2011) it was not strongly associated with treatment effect in our analyses, although higher levels were noted with greater TB extent of disease on baseline chest radiograph. Cathelicidin and vitamin D binding protein (VitD-BP) assays were developed for this study given the important role of Vitamin D processing in TB disease, (Yamshchikov et al., 2010) but we found little association with treatment response for these markers. The biomarkers that had the strongest associations with week-8 culture status, SAA1, CRP, IL1β, IL6, MMP-8 and IFN-γ, overlapped with the biomarkers that had the strongest associations with baseline disease status, as well as the largest changes with treatment. However, of these six biomarkers only three, SAA1, CRP and IL-1β, had associations that met our criteria for significance, yet multivariate regression models, employing multiple biomarkers, did not identify combinations with high predictive utility for week-8 culture conversion. Moreover, analysis of biomarker associations with treatment failure was not feasible as only three trial participants (1%) failed treatment.

Three biomarkers showed increasing levels during treatment: osteocalcin, MCP-1 and MCP-4. The increase in MCP-1 with treatment has been previously noted (Djoba Siawaya et al., 2009). The reported role of MCP-1 and MCP-4 (or monocyte chemoattractant proteins 1 and 4) as attractants for monocytes and T cells, cell types involved in granuloma formation, provides a plausible mechanism by which levels of these chemokines increase in response to treatment (Saunders and Britton, 2007). The increase in osteocalcin with TB treatment is a new finding, however, and the mechanism for this relationship is not clear. Osteocalcin is an osteoblast-specific bone matrix protein involved in bone formation, and has been found to be involved in insulin regulation, (Wei and Karsenty, 2015) suggesting that osteocalcin may play a role in metabolic control during TB infection. Alternatively, osteocalcin levels may be related to pathogen clearance (Das et al., 2013, Garhyan et al., 2015). This is conceivable given reports that *M. tuberculosis* survival post-therapy may involve infection of CD271(+) bone marrow-mesenchymal stem cells, which may act as a protective intracellular niche for *M. tuberculosis* persistence, and given that toll-like receptor stimulation of osteoclast precursors inhibit their differentiation into non-inflammatory mature osteoclasts during microbial infection, instead maintaining the phagocytic activity of the precursor cells (Takami et al., 2002).

Our study has limitations. First, long-term outcomes of interest (relapse versus durable cure) were not a component of the parent phase 2 trial, and our analyses were focused on biomarker associations with baseline severity and microbiologic indicators of treatment effect only. An evaluation of the most promising biomarkers we discovered should be pursued as part of clinical trials with long-term follow-up. Second, we were limited in our ability to explore the dynamics of biomarker change given two time points for serum collection. An assessment of the best performing markers in our study, across additional time points would improve our understanding of the predictive performance of these biomarkers. Third, the parent trial allowed for TB treatment prior to the randomization visit when serum was collected and study drugs initiated. This may have impacted baseline biomarker levels, however, in sensitivity analyses limited to participants with no pre-treatment, the biomarkers with the strongest associations with baseline severity and treatment effect remained the same.

In conclusion, across 70 host biomarkers evaluated in a large and diverse population of TB patients enrolled in a clinical trial, we found that several previously reported putative TB-associated biomarkers (HOMX1, neopterin, and cathelicidin) were not significantly associated with treatment response. However, across numerous analyses we did identify seven recurring biomarkers that 1) were associated with baseline disease severity, 2) were strongly modulated by TB treatment, and 3) had treatment responses that were greater for patients that were culture-converted at 8 weeks. These recurring biomarkers included acute phase proteins (SAA1, PTX-3, PCT and CRP); inflammatory cytokines (IL-1β and IL-6); and a factor associated with tissue reorganization (MMP-8). These biomarkers may provide blood-based targets both for determining baseline disease severity and for monitoring disease during treatment; they warrant examination in patient cohorts with follow-up that captures long-term outcomes of failure and relapse.

## Author Contributions

Conception and design: PN, GBS, AM, and MRS. Data acquisition and management: MRS, LJ, MW, and WCW. Assay development and biomarker measurements: GBS, AM, MW, SB, NS, KH. Analysis and interpretation: PN, GBS, MRS, JLD, MW, JJ, JS, and DL. Drafting the manuscript for important intellectual content: GBS, AM, MRS, DML and PN.

## Disclaimer

References in this manuscript to any specific commercial products, process, service, manufacturer, or company does not constitute its endorsement or recommendation by the U.S. Government or CDC. The findings and conclusions are those of the authors and do not necessarily represent the views of the Centers for Disease Control and Prevention.

## Appendix A Supplementary data

Supplementary material Image 1
