# Supplementary material for: Biomarkers of Tuberculosis Severity and Treatment Effect: A Directed Screen of 70 Host Markers in a Randomized Clinical Trial
Source: eBioMedicine. 2017 Oct 24;25:112–21. doi: 10.1016/j.ebiom.2017.10.018 (PMC5704068; doi:10.1016/j.ebiom.2017.10.018)
Supplement: Supplementary file 1 — Supplementary material [file mmc1.docx]

**Online Supplementary Materials**

**Biomarkers of Tuberculosis Severity and Treatment Effect: A Directed Screen of 70 Biomarkers of Host Response to Infection or Injury in a Randomized Clinical Trial**

**Assay Methods:**

In total, 14 biomarker panel assays were tested, six MSD commercial kits and eight custom assay panels that were newly developed for this study. Six kits selected from the commercially available catalog of MSD assays and run according to package inserts included: 1) V-PLEX® Human Pro-inflammatory Panel 1 (“PP1”: IL-1β, IL-2, IL-4, IL-6, IL-8, IL-10, IL-13, IL-12p70, TNF-α, IFN-γ); 2) V-PLEX Human Cytokine Panel 1 (“CyP1”: GM-CSF, IL-1α, IL-5, IL-7, IL-12(total), IL-15, IL-16, IL-17, TNF-β, VEGF); 3) V-PLEX Human Chemokine Panel 1 (“ChP1”: Eotaxin, Eotaxin-3, IL-8, IP-10, MCP-1, MCP-4, MDC, MIP-1α, MIP-1β, TARC); 4) V-PLEX Human Vascular Injury Panel 2 (“VIP2”: CRP, SAA1, sICAM-1, sVCAM-1); 5) Human Vascular Injury Panel 1 (“VIP1”: E-selectin, P-selectin, thrombomodulin, sICAM-3); and 6) Human LBP assay (“LBP”: LBP). The eight custom assay panels included: 1) TB Panel 1 (“TB1”: Granzyme B, I-TAC, IFN-2α, IFN-β, IL-12p40, MIP-3α, PCT); 2) TB Panel 2 (“TB2”: sIL-2R, Osteocalcin, Osteopontin, Pentraxin-3, sTNF-R1, uPAR); 3) TB Panel 3 (“TB3”: RANTES, sIL-6R, sTNF-R2); 4) TB Panel 4 (“TB4”: MMP-1, MMP-3, MMP-8, MMP-9); 5) TB Panel 5 (“TB5”: CD-40L, Granzyme A, Heme Oxygenase 1 (HOMX-1), IL-22, LAG-3, Neopterin); 6) TB Panel 6 (“TB6”: CD40, CXCL9, TREM); TB Panel 7 (“TB7”: α-Defensin (including α-Defensins 1 through 3) , Cathelicidin) and 8) TB Panel 8 (“TB8”: Vitamin D binding protein (VitD-BP)). These panels included custom multiplexed arrangements of antibody sets available in other MSD kits as well as assays specifically developed for this study (Granzyme B, sIL-2R, Pentraxin-3, uPAR and MMP-8 and all the assays in TB Panels 5 to 8). All custom panels were characterized to establish limits of detection (LOD) and lower and upper limits of quantitation (LLOQ and ULOQ), and to confirm that the assays met pre-set performance specifications for precision (coefficient of variation (CV) < 20% in quantitation range), dilution linearity and spike recovery (concentration between 80% to 120% of expected value) and cross-reactivity (< 0.5 % between assays in a panel).

Assay protocols

The different assay panels were run using the same protocol with sample volumes and dilutions adjusted as needed for each panel. Using the Human Vascular Injury Panel 1 (VIP1) as an example: (1) a VIP1 assay plate was blocked by adding MSD Blocker A to each well, incubating for 0.5 to 1 h and washing 3x with PBS-Tween (PBS-T); (2) 25 µL of VIP1 assay diluent was added to each well followed by 25 µL of pre-diluted sample (diluted 1:10 in VIP1 assay diluent); (3) the plate was incubated for 2 h on a shaker and washed 3x with PBS-T; (4) 25 µL of detection antibody mixture (in VIP1 antibody diluent) was added; (5) the plate was incubated for 1 h on a shaker and washed 3x with PBS-T; and (6) 150 µL of MSD Read Buffer T was added to each well and the plate was read on an MSD SECTOR S 600 ECL plate reader. The other panels were run in a similar manner, but sample dilutions were adjusted as follows: no dilution (PP1, CyP1, TB1, TB5), 1:2 dilution (ChP1), 1:5 dilution (TB6), 1:10 dilution (VP1, TB2), 1:20 dilution (TB4), 1:200 dilution (VIP2, LBP, TB3), 1:500 dilution (TB7), and 1:500,000 dilution (TB8).

For quantification of analyte concentrations, each plate included a seven-point calibration curve (prepared by serial dilutions of the combined calibration standard) and a blank run in duplicate. To assess plate-to-plate consistency over the course of the screen, each plate included duplicate measurements of a QC sample that had measurable levels of all analytes in the multiplex panel. Up to 39 samples could be run, in duplicate, in the remaining wells. The MSD instrument software, DISCOVERY WORKBENCH^®^, was used to calculate concentrations for the control and unknown samples based on a four parameter logistic (4-PL) fit to the calibration data.

**Supplementary Table 1.** Distribution of biomarker levels (pg/mL) across all samples

|  |  | **Quantile** | | |  |  |  | **Quantile** | | |
| --- | --- | --- | --- | --- | --- | --- | --- | --- | --- | --- |
| **Assay** | **LOD** | **25%** | **50%** | **75%** |  | **Assay** | **LOD** | **25%** | **50%** | **75%** |
| α-Defensin | 150000 | 1700000 | 2700000 | 4100000 |  | IP-10 | 0.34 | 390 | 700 | 1500 |
| Cathelicidin | 130000 | 1000000 | 1400000 | 1900000 |  | LAG-3 | 6.6 | 630 | 1100 | 1800 |
| CD40 | 4.3 | 140 | 170 | 210 |  | LBP | 13000 | 4.1x10^5^ | 6.8x10^6^ | 1.1x10^7^ |
| CD40L | 8.4 | 1000 | 1700 | 2400 |  | MCP-1 | 0.3 | 150 | 200 | 280 |
| CRP | 1900 | 1.1x10^7^ | 4.5x10^7^ | 1.2x10^8^ |  | MCP-4 | 6.5 | 90 | 130 | 170 |
| CXCL9 | 0.36 | 100 | 210 | 420 |  | MDC | 8.8 | 910 | 1100 | 1500 |
| E-Selectin | 460 | 19000 | 28000 | 40000 |  | MIP-1a | 3 | 15 | 23 | 38 |
| Eotaxin | 4.5 | 55 | 76 | 120 |  | MIP-1b | 0.63 | 99 | 130 | 170 |
| Eotaxin-3 | 6.9 | 12 | 16 | 22 |  | MIP-3a | 3.6 | 23 | 41 | 77 |
| GM-CSF | 0.5 | 0.5 | 0.5 | 0.5 |  | MMP-1 | 76 | 21000 | 39000 | 66000 |
| Granzyme-A | 10 | 39 | 61 | 92 |  | MMP-3 | 94 | 12000 | 19000 | 27000 |
| Granzyme-B | 2.5 | 63 | 91 | 140 |  | MMP-8 | 36 | 18000 | 35000 | 74000 |
| HOMX-1 | 93 | 370 | 540 | 880 |  | MMP-9 | 5900 | 460000 | 770000 | 1300000 |
| I-TAC | 1.1 | 160 | 380 | 990 |  | Neopterin | 28 | 40 | 50 | 66 |
| IFN-2α | 0.89 | 0.89 | 0.89 | 0.99 |  | Osteocalcin | 410 | 33000 | 52000 | 74000 |
| IFN-β | 23 | 23 | 23 | 23 |  | Osteopontin | 210 | 23000 | 31000 | 42000 |
| IFN-γ | 0.63 | 15 | 37 | 84 |  | P-Selectin | 150 | 86000 | 110000 | 130000 |
| IL-10 | 0.08 | 0.23 | 0.38 | 0.68 |  | PCT | 1.1 | 12 | 20 | 36 |
| IL-12-p40 | 5.2 | 18 | 26 | 40 |  | PTX-3 | 160 | 3100 | 5300 | 9600 |
| IL-12-p70 | 0.17 | 0.17 | 0.17 | 0.17 |  | RANTES | 410 | 200000 | 350000 | 540000 |
| IL-12-Total | 0.88 | 130 | 190 | 270 |  | SAA1 | 15000 | 4.1x10^6^ | 2.9x10^7^ | 1.2x10^8^ |
| IL-13 | 1.1 | 1.1 | 1.1 | 1.1 |  | sICAM-1 | 840 | 450000 | 590000 | 750000 |
| IL-15 | 0.53 | 2.3 | 3.0 | 3.8 |  | sICAM-3 | 19 | 770 | 980 | 1300 |
| IL-16 | 2.2 | 150 | 200 | 260 |  | sIL-6R | 49 | 27000 | 32000 | 41000 |
| IL-17 | 1.4 | 1.5 | 2.4 | 3.8 |  | sVCAM-1 | 16000 | 470000 | 580000 | 740000 |
| IL-1α | 0.3 | 0.3 | 0.3 | 0.3 |  | TARC | 1.8 | 320 | 490 | 820 |
| IL-1β | 0.05 | 0.047 | 0.11 | 0.33 |  | Thrombomodulin | 110 | 3600 | 4300 | 5200 |
| IL-2 | 0.15 | 0.15 | 0.15 | 0.21 |  | TNF-a | 0.24 | 4.2 | 5.7 | 8.2 |
| IL-22 | 0.42 | 2.3 | 4.0 | 7.0 |  | TNF-b | 0.13 | 0.18 | 0.25 | 0.33 |
| IL-2R | 64 | 2700 | 3900 | 5600 |  | TNF-RI | 34 | 2300 | 3000 | 4000 |
| IL-4 | 0.03 | 0.027 | 0.027 | 0.033 |  | TNF-RII | 17 | 5200 | 7100 | 9900 |
| IL-5 | 0.25 | 0.25 | 0.38 | 0.84 |  | TREM | 15 | 240 | 330 | 450 |
| IL-6 | 0.1 | 1.0 | 2.6 | 7.0 |  | uPAR | 18 | 4100 | 5200 | 7000 |
| IL-7 | 0.28 | 21 | 31 | 42 |  | VEGF | 0.73 | 89 | 170 | 350 |
| IL-8 | 0.08 | 11 | 14 | 22 |  | VitD-BP | 2.0x10^6^ | 7.4x10^8^ | 8.7x10^8^ | 1.0x10^9^ |

LOD is the limit of detection for the biomarker assay*.*

**Supplementary Table 2.** Effect of TB treatment on biomarker levels.

|  | **C(Week 8) / C(Week 0)** | | | |
| --- | --- | --- | --- | --- |
| **Assay** | **Geom. Mean (95%CI)** | **log(p value)** | **Median (IQR)** | **% Aligned** |
| **SAA1** | 0.10 (0.09, 0.12) | -82 | 0.10 (0.03, 0.33) | 94% |
| **CRP** | 0.19 (0.17, 0.22) | -73 | 0.20 (0.09, 0.47) | 94% |
| **IL-6** | 0.28 (0.25, 0.31) | -70 | 0.29 (0.14, 0.54) | 90% |
| **MMP-8** | 0.37 (0.33, 0.41) | -58 | 0.37 (0.22, 0.63) | 89% |
| **IL-1β** | 0.44 (0.40, 0.49) | -39 | 0.47 (0.24, 1.00) | 73% |
| **IFN-γ** | 0.45 (0.40, 0.50) | -36 | 0.44 (0.21, 0.82) | 81% |
| **LBP** | 0.47 (0.44, 0.49) | -87 | 0.46 (0.34, 0.65) | 94% |
| **I-TAC** | 0.48 (0.44, 0.52) | -47 | 0.48 (0.30, 0.71) | 87% |
| **IL-22** | 0.49 (0.45, 0.53) | -50 | 0.51 (0.30, 0.77) | 85% |
| **MMP-1** | 0.49 (0.47, 0.52) | -74 | 0.49 (0.36, 0.68) | 94% |
| **VEGF** | 0.52 (0.48, 0.55) | -59 | 0.54 (0.41, 0.72) | 95% |
| **PCT** | 0.52 (0.47, 0.57) | -33 | 0.60 (0.36, 0.88) | 83% |
| **MMP-9** | 0.52 (0.48, 0.56) | -42 | 0.52 (0.35, 0.75) | 85% |
| **CXCL9** | 0.55 (0.51, 0.60) | -33 | 0.56 (0.35, 0.88) | 81% |
| **PTX-3** | 0.59 (0.55, 0.63) | -37 | 0.60 (0.39, 0.92) | 79% |
| **IP-10** | 0.59 (0.53, 0.66) | -20 | 0.57 (0.36, 0.92) | 80% |
| **Osteocalcin** | 1.63 (1.55, 1.71) | -54 | 1.55 (1.22, 2.00) | 88% |
| **α-Defensin** | 0.62 (0.59, 0.65) | -62 | 0.63 (0.47, 0.80) | 88% |
| **IL-7** | 0.65 (0.63, 0.68) | -60 | 0.66 (0.52, 0.81) | 89% |
| **IL-2R** | 0.73 (0.70, 0.75) | -43 | 0.72 (0.59, 0.89) | 83% |
| **TNF-RI** | 0.73 (0.70, 0.75) | -57 | 0.73 (0.59, 0.89) | 87% |
| **TREM** | 0.76 (0.74, 0.79) | -41 | 0.77 (0.63, 0.91) | 85% |
| **MCP-4** | 1.31 (1.26, 1.37) | -29 | 1.28 (1.01, 1.62) | 76% |
| **MCP-1** | 1.22 (1.18, 1.26) | -23 | 1.19 (1.02, 1.46) | 78% |
| **uPAR** | 0.83 (0.80, 0.85) | -26 | 0.84 (0.70, 0.98) | 78% |
| **sICAM-1** | 0.83 (0.81, 0.85) | -32 | 0.82 (0.70, 0.95) | 82% |

C(Week 8) / C(Week 0) is the ratio of biomarker levels after and before treatment. Only biomarkers with an IQR that does not include the value 1 (no change on treatment) are shown.

The mean value is the geometric mean of the log transformed biomarker level. The p value is determined by t test (two-sided, p < 0.05 / 62 assays = 0.0008 taken as statistically significant, with Bonferroni correction to account for multiple testing. % Aligned is a non-parametric indicator of effect size and indicates the percentage of points with the same directionality of effect as the mean value. A higher value corresponds to a more consistent effect across the patient population and a higher predictive value.

**Supplementary Table 3.** Association of biomarker treatment effect with week 8 culture status

|  | **Unadjusted Model** | | | **Adjusted (Demographics)** | | | **Adjusted (Baseline Disease)** | | |
| --- | --- | --- | --- | --- | --- | --- | --- | --- | --- |
| **Assay** | **Est (95%CI)** | **P Value** | **AUC** | **Est (95%CI)** | **P Value** | **AUC** | **Estf (95%CI)** | **P Value** | **AUC** |
| **SAA1** | 1.81 (1.51, 2.16) | 0.0009 | 0.62 | 1.96 (1.63, 2.35) | 0.0003 | 0.64 | 1.98 (1.62, 2.41) | 0.0006 | 0.64 |
| **CRP** | 1.76 (1.53, 2.03) | 0.0001 | 0.65 | 1.67 (1.44, 1.93) | 0.0005 | 0.63 | 1.59 (1.36, 1.85) | 0.0026 | 0.63 |
| **IL-1b** | 1.25 (1.11, 1.40) | 0.0496 | 0.56 | 1.40 (1.25, 1.57) | 0.0037 | 0.61 | 1.55 (1.37, 1.75) | 0.0004 | 0.63 |
| **IL-6** | 1.24 (1.11, 1.39) | 0.0587 | 0.56 | 1.35 (1.21, 1.52) | 0.0092 | 0.60 | 1.47 (1.30, 1.65) | 0.0019 | 0.62 |
| **IFN-g** | 1.22 (1.08, 1.37) | 0.0936 | 0.55 | 1.22 (1.08, 1.38) | 0.1004 | 0.55 | 1.21 (1.06, 1.38) | 0.1543 | 0.55 |
| **MMP-8** | 1.11 (1.00, 1.23) | 0.3218 | 0.54 | 1.23 (1.10, 1.37) | 0.0557 | 0.58 | 1.32 (1.17, 1.49) | 0.0183 | 0.60 |

The estimated association (Est) is 10^coeff^, where coeff is the coefficient of a linear model for the effect of week 8 culture status on log_10_ transformed concentration ratios. The value can be interpreted as the expected factor increase in the ratio of week 8 and week 0 biomarker levels for non-converters relative to converters. The table shows values for an unadjusted model as well as a model that is adjusted for demographic covariates (gender, age, BMI, HIV status, region (Africa vs. Not-Africa) and study arm), and a model that includes the demographic covariates as well as additional covariates related to disease status at baseline. The top three biomarkers had statistically significant associations (based on the p value) in at least one model. The remaining three biomarkers are those that had a strong response to treatment (Supplemental material Table 2 Effect of TB treatment on biomarker levels) and an observed association with week 8 culture status, but did not achieve our threshold for significance (two-sided t-statistic, p < 0.05 taken as statistically significant, with Bonferroni correction to account for multiple testing). The AUC value for ROC curves compares the distributions of biomarker ratios for the two outcome classes (either unadjusted or after adjusting the biomarker ratios for the covariates).
